# A novel asymmetric 3D in-vitro assay for the study of tumor cell invasion

**DOI:** 10.1186/1471-2407-9-415

**Published:** 2009-11-30

**Authors:** Vera Brekhman, Gera Neufeld

**Affiliations:** 1 Cancer Research and vascular Biology Center, The Bruce Rappaport Faculty of Medicine, Technion, Israel Institute of Technology, PO Box 9679, 1 Efron St, Haifa, 31096, Israel

## Abstract

**Background:**

The induction of tumor cell invasion is an important step in tumor progression. Due to the cost and slowness of *in-vivo *invasion assays, there is need for quantitative *in-vitro *invasion assays that mimic as closely as possible the tumor environment and in which conditions can be rigorously controlled.

**Methods:**

We have established a novel asymmetric 3D in-vitro invasion assay by embedding a monolayer of tumor cells between two layers of collagen. The cells were then allowed to invade the upper and lower layers of collagen. To visualize invading cells the gels were sectioned perpendicular to the monolayer so that after seeding the monolayer appears as a thin line precisely defining the origin of invasion. The number of invading tumor cells, their proliferation rate, the distance they traverse and the direction of invasion could then be determined quantitatively.

**Results:**

The assay was used to compare the invasive properties of several tumor cell types and the results compare well with those obtained by previously described assays. Lysyl-oxidase like protein-2 (Loxl2) is a potent inducer of invasiveness. Using our assay we show for the first time that inhibition of endogenous Loxl2 expression in several types of tumor cells strongly inhibits their invasiveness. We also took advantage of the asymmetric nature of the assay in order to show that fibronectin enhances the invasiveness of breast cancer cells more potently than laminin. The asymmetric properties of the assay were also used to demonstrate that soluble factors derived from fibroblasts can preferentially attract invading breast cancer cells.

**Conclusion:**

Our assay displays several advantages over previous invasion assays as it is allows the quantitative analysis of directional invasive behavior of tumor cells in a 3D environment mimicking the tumor microenvironment. It should be particularly useful for the study of the effects of components of the tumor microenvironment on tumor cell invasiveness.

## Background

The transition from the non-invasive phenotype to the invasive phenotype of tumor cells marks the transition from a relatively benign tumor that can be successfully treated surgically to a more malignant form of cancer [1]. Despite many in vivo studies of tumor cell invasion, the amount of information derived from such in vivo studies is somewhat limited due to the complex microenvironment of tumors, due to the inherent variability frequently encountered in in-vivo assays, and due to the cost of in-vivo assays. This problem can be partially overcome by studying invasive processes in relatively inexpensive in-vitro tumor progression assays in which conditions can be accurately controlled. However, for meaningful results such in-vitro systems should mimic as closely as possible in-vivo conditions.

In-vitro 3D invasion assays can be divided into three general types. The first is represented by Boyden chamber and similar filter-based invasion assays in which tumor cells are seeded on top of a gel composed of extracellular matrix (ECM)-derived proteins which sits on top of a filter. The cells are allowed to invade and cells that pass to the other side of the filter are counted [2,3]. The advantage of this type of assay is speed and ease of quantitative determination. However, only the end point is monitored, the cells that successfully invaded usually represent only a small fraction of the entire population of tumor cells and may only represent a subpopulation of the tumor cells. Thus, results obtained in such assays may be misleading. In the second type of assay cells are seeded as suspensions in gels composed of ECM components and their migration in the gel is monitored [4,5]. Alternatively, the tumor cells are pre-formed into spheroids which are subsequently suspended in the gels or seeded on top of gels. The migration of the implanted cells out of the spheroids is then monitored [6-9]. These types of assays are much slower and are generally difficult to quantify because of the absence of a sharply defined origin of migration. Thus quantification relies on the tracking of individual cells within the population or on semi-quantitative assessments of the expansion of implanted spheroids. In the third type of assay, cells are seeded on top of a gel composed of ECM components. The cells adhere forming a monolayer and subsequently invade the gel. This is very similar to the Boyden chamber based assays except that in these assays various methods are used to track invading cells and the assays do not simply rely on the counting of cells able to traverse a filter [10,11]. In these assays the start position is clearly defined by the upper surface of the gel aiding quantification and the whole population of cells can be observed but the assays take much more time as compared with Boyden chamber based assays. A major problem of this type of assay is that at the start position cells are located at an interface between liquid and gel and are not embedded completely as they embedded in-vivo. Furthermore, this assay is unidirectional, and it is therefore difficult to compare the effects of different external factors on the invasive properties of the tumor cells.

We describe a novel 3D invasion assay in which in the initial "start" position tumor cells are seeded in a monolayer between two gel layers. In this assay the cells are completely embedded in the ECM from the start. The effects of various constituents of the tumor microenvironment on the invasive behavior of tumor cells can be compared by differentially varying the composition of the microenvironment that tumor cells encounter above or below the tumor cell monolayer. Thus, it is possible, for example, to embed different types of normal cells in the ECM above or below the monolayer of tumor cells and directly compare their effects on tumor cell invasiveness in a single experiment. Furthermore, since at the beginning of the assay the cells are arranged in a monolayer that defines a sharp origin of migration, this assay is also easily quantifiable and provides information on behavior of the entire population of the tumor cells.

## Methods

### Reagents

The ΔNRF (pCMVdR8.74) and pMD2-VSVG plasmids were given to us by Dr. Tal Kafri (Department of Microbiology and Immunology, University of North Carolina). A human Loxl2 specific shRNA lentiviral plasmid set was purchased from Sigma (MISSION shRNA Bacterial Glycerol Stock NM-002318). Two of the shRNA's (TRCN46195 and TRCN46197) efficiently inhibited Loxl2 expression (data not shown). The MISSION Non-Target shRNA Control Vector was purchased from Sigma (SHC002). The vital fluorescent dyes DiI and DiAsp were purchased from Molecular Probes (Eugene, OR). Eosin-Y was from Pioneer Research Chemicals, UK. Mayer's hematoxylin was from Sigma. Laminin-1 derived from Engelbreth-Holm-Swarm (EHS) tumors was kindly provided by Dr. Hynda Kleinman (NIH, Bethesda, Maryland, USA). Rat tail collagen was also purchased from Trevigen (Gaithersburg, USA).

### Cells

The MDA-MB-231 cells were kindly provided by Dr. Michael Klagsbrun (Harvard University, USA). The LM2-4 cells were provided by Dr. Robert Kerbel from Sunnybrook and Women's College Health Sciences Centre, Toronto, Canada. HT1080 cells were provided to us by Dr. Varda Rotter (Weizmann institute, Israel). The MDA-MB-435 and HEK293-T cells were purchased from the ATCC. Human foreskin fibroblasts were isolated as described [12]. The cells were grown in DMEM supplemented with 4.5 g/ml glucose, 10% FCS, 2 mM Glutamine, 50 μg/ml gentamicin, 2.5 μg/ml fungizone, in a humidified incubator at 37°C, 5% CO_2_.

### Preparation of rat tail collagen

Tails were removed from freshly killed adult rats and were cleaned by brief washing in 70% ethanol. The skin was split at the tail root and peeled away. The distal and proximal quarters of the tail were cut away. Each tendon was separately dissected using a scalpel and the fibers separated. The fibers were rinsed briefly in 70% ethanol, weighted, and incubated in 0.2% acetic acid (100 ml/gr). The flask was stirred three to five days at 4°C. Insoluble debris was separated by centrifugation. The supernatant was aliquoted and stored at 4°C. Collagen concentration was determined by weighing following freeze drying of an aliquot of the supernatant. Similar results were obtained using a commercial preparation of rat tail collagen.

### Invasion assay

Unless otherwise stated, the lower collagen layer was prepared by mixing one volume of tenfold concentrated M199 growth medium, one volume of 1 mg/ml bovine fibronectin (final concentration 0.1 mg/ml), 0.1 volume of 10% FCS (final concentration 1%) and seven volumes of collagen type I solution (final concentration 1 mg/ml). 2 N NaOH was then added slowly while mixing to bring the pH to pH-7.3 to obtain a neutralized solution of collagen. All the constituents were kept at 4°C as was the final solution. Cold collagen solution (0.3 ml) was poured into 8-wells chamber wells (Nunc) avoiding bubble formation and warmed to 37°C to induce polymerization.

Cancer cells were trypsinized and suspended in complete growth medium. 200 μl containing 5 × 10^4 ^cells were seeded on the top of the lower collagen layer and allowed to adhere and spread. After two hours, the medium was aspirated gently and unless otherwise stated, 0.2 ml of the same cold neutralized collagen solution was added to cover the cells and to form the upper layer of collagen. The upper layer was then polymerized at 37°C. For tumor cells/fibroblasts mixed assays an additional layer of 0.12 ml collagen containing 10^5 ^normal human skin fibroblasts (HSF) was formed on top of the upper collagen layer. Following polymerization, 0.25 ml of growth medium was added to the wells and the cells were incubated for up to 9 days in a humidified incubator at 37°C and 5% CO_2_. The medium covering the collagen gel was exchanged every two days.

### Preparation of histological sections from gels

**Figure 1:**
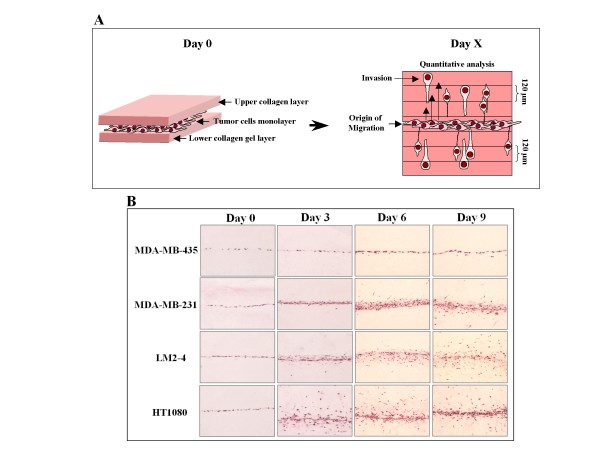
**Establishment of the invasion assay and comparison of the invasive potential of different tumor cell types**: **A**. In order to assay differences in the invasive potential of different types of tumor cells, the tumor cells are seeded at day 0 on top of a gel composed of collagen. Following cell adhesion and spread a second layer of collagen is seeded on top of the cell monolayer. The gels are subsequently cultured between 3 and 9 days and invasion of cells from the monolayer into the collagen assayed as described in methods. **B**. The invasive properties of MDA-MB-435 melanoma cells, MDA-MB-231 and LM2-4 breast cancer cells and HT1080 fibrosarcoma cells were compared. Following the seeding of the cells, the invasion of cells from the monolayer into the upper and lower collagen gel was measured in intervals of 3 days. Representative low power (10×) pictures of perpendicular gel sections in which the tumor cells are stained with eosin-Y are shown.

The medium was gently aspirated and the gels were fixed by addition of 0.5 ml of 4% paraformaldehyde in phosphate buffered saline (PBS). The 4% paraformaldehyde solution was exchanged twice at 30 minute intervals. Gels were then washed with PBS three times at 10 minute intervals. The PBS was then gently aspirated and the gels were covered with Tissue Embedding Medium (OCT) and incubated overnight at 4°C. The gels were then transferred to self-prepared aluminum foil molds, embedded with additional OCT and immediately frozen in isopentane pre-cooled by liquid nitrogen. Constructs were then flipped by 90 degrees and cut in a cryostat into 7 μm thick sections in order to display a perpendicular cross section of the cell monolayer (Figures 1A and 1B). Sections were air-dried, rinsed with running water, stained with eosin-Y for 20 seconds, rinsed and counterstained with Mayer's hematoxylin prior to mounting.

### Quantification of invasion

Stained sections were viewed at a 10× magnification and the middle portion of each section was photographed. The "measure" function of the Image-Pro software was used to highlight the cells and to count cells in successive zones 120 μm thick above and below the central line of cells that did not migrated out of the monolayer (Figure 1A)). The number of stained cells larger than 30 pixels in each of the 120 μm wide zones was determined. In symmetrical experiments in which the upper and lower gels were similar, the number of the cells that invaded corresponding zones above and below the monolayer was summed to obtain the number of invading cells in each of the zones.

### Boyden chamber invasion assay

The assay was performed using 24-well Boyden chambers containing polycarbonate filters with a pore size of 8 μm essentially as described [3]. The filters were coated with 50 μl of neutralized collagen I solution, and incubated in a humidified incubator at 37°C to allow polymerization. The lower compartment was filled with DMEM supplemented with 10% fetal calf serum. HT-1080 cells (10^5 ^cells/well) expressing Loxl2 targeting or control shRNAs were suspended in 0.5 ml serum free DMEM and seeded on top of the collagen in the upper compartment of the chamber. After incubation at 37°C for 16 h, the cells on the upper surface of the filter were removed by wiping. Cells, that transversed the filter were fixed in methanol, stained with 0.5% crystal violet, and counted.

### Inhibition of Loxl2 expression in tumor cells

HEK293-T cells were co-transfected with the lentiviral plasmid containing the Loxl2 specific shRNA or with a plasmid containing control unrelated shRNA (8 μg), along with the packaging vector pCMVdR8.91 (5 μg), and a plasmid encoding the vesicular stomatitis virus coat envelope pMD2-VSVG (2 μg) using Fugene-6 according to the instructions of the vendor and incubated 24 h at 37°C. Conditioned medium containing infective lentiviral particles was collected 48 hours and 72 hours post transfection, and filtered through 0.45 μ filters and used for infection of cells. Target cells (10^5 ^cells) were incubated with the conditioned medium in the presence of 8 μg/ml polybrene for 8-16 hours. Following infection, cells were washed twice with PBS, transferred to the regular growth medium. Stable Loxl2-silenced cells were selected using 2 μg/ml puromycin. To verify Loxl2 down-regulation, cells were lysed 48-72 hours post infection. Cell lysates (30 μg) were separated by SDS/PAGE, blotted onto nitrocellulose, and probed with antibodies directed against Loxl2 [13].

### Fluorescent labeling of cells

In assays in which both tumor cells and fibroblasts were used, the tumor cells were labeled with 5 μg/ml DiI (green fluorescence) by incubation in complete growth medium for 0.5 h prior to the assay. The human skin derived fibroblasts (HSF cells) were labeled similarly with 5 μg/ml DiAsp (red fluorescence).

### Statistical analysis

At least three independent experiments were assessed. Cells in eight to twelve microscopic fields were counted in each experiment. Error bars represent the standard error of the mean. The statistical significance of the results was examined using the unpaired data with unequal variance student's T-test. Statistical significance is presented in the figures in the following way: *p < 0.05, **p < 0.01 and ***p < 0.001.

## Results

### Establishment of the invasion assay and comparison of the invasive properties of different tumorigenic cell types

Tumor cells were seeded on top of a pre-formed rat tail collagen gel and allowed to adhere. In previously described 3D invasion assays the tumor cells are then allowed to invade the underlying layer of collagen [10]. However, in order to mimic the in-vivo conditions in which the tumor cells are completely embedded in tissue, we poured a second layer of collagen on top of the monolayer of the tumor cells. In essence, the resulting monolayer of tumor cells can be viewed as a two dimensional tumor that is bordered on each side by a different tissue. When the completed two layer gels were sectioned perpendicularly to the tumor cell monolayer shortly after the seeding of the cells, the monolayer was seen as a thin line of cells that clearly defines an origin of migration or start position (Figures 1A and 1B, Day 0).

To compare the invasive properties of different types of tumor cells we used MDA-MB-435 cells, which are well differentiated human melanoma cells that still express many differentiated markers of melanocytes [14], MDA-MB-231 breast cancer derived cells and MDA-MB-231 derived LM2-4 cells which differ from the parental cell line by their increased lung metastases forming ability [15]. Lastly, we used highly invasive fibrosarcoma derived HT1080 cells that are generally considered to be among the most invasive tumor cell types [16,17].

**Figure 2:**
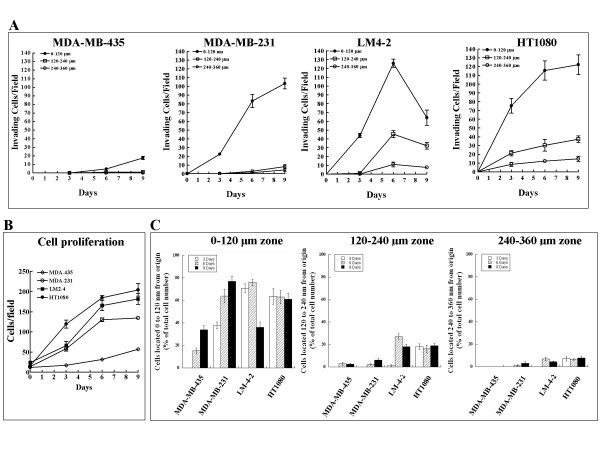
**Quantitative analysis of the tumor cells invasion assay shown in Figure 1**. **A**. The area above and below the cell monolayer was divided into three subsequent 120 μm wide zones as shown in Figure 1A. The number of invading cells in each of the zones was determined at three day intervals as described in methods. Each point represents the average number of cells counted in at least eight different microscopic fields derived from three separate experiments. **B**. The average number of each of the tumor cell types was determined as a function of time following monolayer formation in microscopic fields using the image-pro software as described in methods. **C**. The number of cells that have invaded each of the zones as a function of time is presented here as a percentage out of the total number of cells. Error bars represent the standard error of the mean.

Of these cell lines, the HT1080 cells exhibited, as expected, the highest invasion rate in our assay while the MDA-MB-435 cells appeared to be the least invasive (Figures 1B and 2A). As predicted, parental MDA-MB-231 cells were less invasive than their highly metastatic LM2-4 offspring (Figures. 1B and 2A). The HT1080 and the LM2-4 cells were clearly much more invasive than the MDA-MB-435 and MDA-MB-231 cells, as evidenced by the number of cells that successfully migrated more than 120 μm into the surrounding collagen layers after 6 days (Figures. 1B and 2A). Unlike Boyden chamber assays, which are usually short term assays, here cells are allowed to invade and migrate over a period of several days. This is long enough to allow significant cell proliferation, and should be controlled for since the invading cells also proliferate. Indeed, the rate of proliferation correlated with the invasive potential of the cells. HT1080 cells proliferated at the fastest rate while the proliferation of MDA-MB-435 cells was the slowest (Figure 2B). To compensate for the proliferation, we assumed that the rate of proliferation of the whole cell population, both of cells that did invade and those that stayed at the origin was similar. We then divided the number of the invaded cells in each of the zones by the total number of cells to obtain the percentage of the entire cell population in each of the zones. By this criteria too HT1080 and LM2-4 cells are the most invasive as demonstrated by their ability to traverse longer distances in the gels at a faster rate (Figure 2C). Interestingly, In the case of the HT1080 cells, the percentage of cells which have invaded distant zones was the same after 3 days or after 9 days (Figure 2C), suggesting that there may exist a subpopulation of cells that migrates very rapidly while another subpopulation does not migrate as robustly, and that all the cells found at distant locations are derived from this rapidly migrating subpopulation.

### Inhibition of Loxl2 expression in several invasive tumor cell types inhibits invasiveness

**Figure 3:**
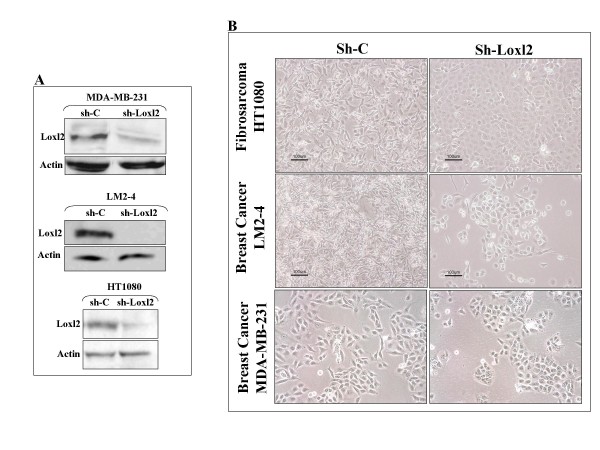
**Inhibition of Loxl2 expression in tumor cells induces transition from a mesenchymal to epithelial like morphology**. **A**. The expression of Loxl2 in MDA-MB-231 and LM2-4 breast cancer cells and HT1080 fibrosarcoma cells was inhibited following infection with lentiviruses producing the Loxl2 targeting shRNA TRCN46195 (sh-195). Control cells were infected with a lentiviral vector carrying the control unrelated shRNA SHC002 (sh-c). Shown are western blots of cell lysates derived from control versus sh-195 infected cells probed with a polyclonal antibody directed against Loxl2 [13]. **B**. Phase contrast microscopy pictures of cultures of HT1080, LM2-4 and MDA-MB-231 cells stably expressing the control shRNA SHC002 or the Loxl2 silencing shRNA TRCN46195.

**Figure 4:**
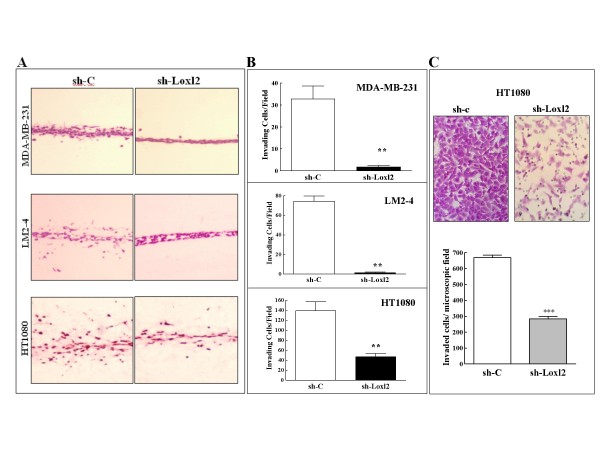
**Comparison of the 3D invasion assay and the Boyden chamber invasion assay**. **A**. MDA-MB-231, LM2-4 and HT1080 cells infected with lentiviruses encoding the control shRNA SHC002 (sh-control) or lentiviruses encoding the Loxl2 specific shRNA TRCN46195 (sh-Loxl2) were seeded as monolayers in collagen as described under Figure 1. After 4 days the gels frozen and were sectioned perpendicular to the cell monolayer. Shown are cryostat sections in which the cells were stained with eosin-Y. Three independent experiments were performed with similar results. **B**. The number of tumor cells infected with a control shRNA or with the Loxl2 specific shRNA encoding lentiviruses was determined as described in methods. Each histogram represents the average number of invading cells determined from examination of eight to twelve microscopic fields derived from three different experiments. **C**. Representative pictures of HT1080 fibrosarcoma cells infected with lentiviruses encoding control (sh-control) or Loxl2 targeting (sh-Loxl2) shRNA species that have invaded and crossed the filter of Boyden chambers is shown. The histogram shows the average number of cells that transversed the Boyden chamber filter in two independent experiments. Error bars represent the standard error of the mean. *** p < 0.001.

Loxl2 is a member of the lysyl-oxidase family of enzymes. We have previously characterized it as a promoter of tumor cell invasiveness [13]. The increased invasiveness was subsequently attributed to Loxl2 induced inhibition of E-cadherin expression in tumor cells [18,19]. Inhibition of Loxl2 expression in highly invasive HT1080 and LM2-4 cells as well as in MDA-MB-231 cells using a specific shRNA species directed against Loxl2 (Figure 3A) induces morphological changes resembling mesenchymal to epithelial transition (Figure 3B). These changes were accompanied by very strong inhibition of the invasive ability of these three cell types (Figure 4A and 4B) indicating that the assay faithfully correlates with in-vivo observations and confirming that up-regulation of Loxl2 in tumor cells is in all likelihood a major cause of enhanced tumor cell invasiveness. To verify these results by a different assay we measured the effects of the inhibition of Loxl2 expression on the invasiveness of HT1080 cells using a Boyden chamber invasion assay. In the Boyden chamber assay too we found that inhibition of Loxl2 expression strongly inhibits the invasiveness of the HT1080 cells (Figure 4C).

### The composition of the extracellular matrix has a major effect on tumor cell invasiveness

**Figure 5:**
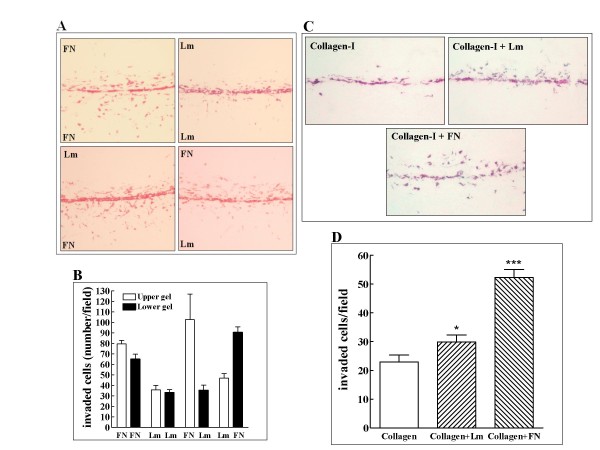
**Comparison of the effects of fibronectin and laminin on tumor cells invasiveness**. **A**. LM-2-4 cells (6 × 10^4^cells/well) were seeded between two collagen layers as described in methods. Laminin (0.1 mg/ml) or fibronectin (0.1 mg/ml) were selectively added to the upper or lower collagen layers or to both collagen layers. The experiment was terminated after 3 days and invading cells stained in gels as described. **B**. LM2-4 cells that invaded the upper or lower collagen gels were counted as described in methods. Shown are the results of two independent experiments. At least twelve microscopic fields were counted to obtain individual average data points. **C**. The effects of laminin and fibronectin on the ability to invade collagen gels were measured as described under A. **D**. Quantification of the relative effects of laminin and fibronectin on the invasiveness of LM2-4 cells was performed as described under B. Error bars represent the standard error of the mean. *p < 0.05, *** p < 0.001.

Invasive tumor cells migrate through tissues that differ in the composition of their ECM. Fibronectin and laminin are extracellular adhesion molecules that associate with collagen [20]. We have utilized the asymmetric character of our invasion assay in order to compare the effects of fibronectin and laminin on tumor cell invasiveness. We added laminin or fibronectin selectively to the upper or lower collagen layers or to both layers and examined the relative effects of the adhesion molecules on the invasive behavior of LM2-4 breast cancer cells. The LM2-4 cells migrated vigorously into collagen that contained fibronectin but invaded much less successfully into collagen layers containing laminin. Thus, when the upper collagen layer contains fibronectin and the lower laminin, the cells migrated more aggressively into the upper gel layer and vice versa (Figures 5A and 5B). Interestingly, laminin also acts as an inducer of tumor cell invasion although it is less potent than fibronectin, since the LM2-4 cells invade laminin containing collagen more efficiently than collagen that does not contain any added adhesion molecules (Figures 5C and 5D).

**Figure 6:**
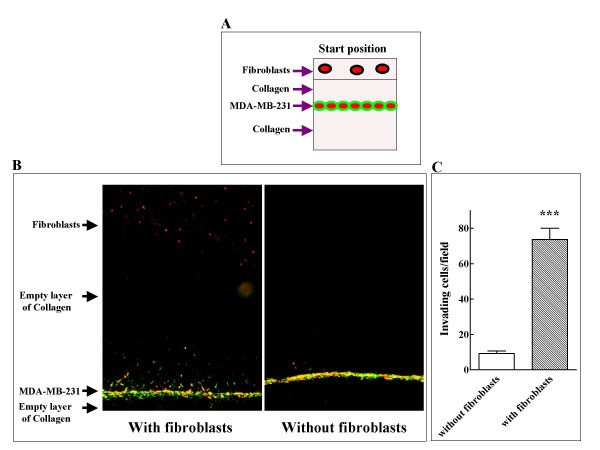
**Fibroblast derived soluble factors attract MDA-MB-231 breast cancer cells and enhance their invasiveness**. **A**. Schematic representation of the 3D invasion assay shown in panel B. **B**. MDA-MB-231 cells were labeled with DiI (green fluorescence) and normal fibroblasts labeled with DiAsp (red fluorescence). Tumor cells were seeded in collagen gels in the presence or absence of fibroblasts as described in methods. Shown is a perpendicular section of a representative collagen gel examined with a fluorescent microscope. **C**. Quantification of tumor cells induced to invade by fibroblasts was performed as described under Figure 2.

Tissues also contain many different types of normal cells which can in turn affect the behavior of invading tumor cells. Once again we took advantage of the asymmetric nature of the assay to set up an experiment designed to determine if fibroblasts secrete attractive factors that enhance tumor cell invasiveness. We labeled MDA-MB-231 breast cancer cells which normally do not display a highly invasive behavior (Figure 1B) with a fluorescent dye and we then embedded a monolayer of these cells in collagen. Above the empty upper layer of collagen we formed another layer of collagen that contained a suspension of normal human foreskin fibroblasts labeled with a different fluorescent dye as depicted (Figure 6A). The cells were allowed to migrate for four days and perpendicular sections of the gels were then observed by fluorescent microscopy. It can be seen that the tumor cells migrated preferentially towards the fibroblasts suggesting that fibroblasts secrete attractive factors that are sensed by the breast cancer cells and induce directional invasion towards the fibroblasts. In contrast, in the absence of fibroblasts there was much less invasion (Figure 6B and 6C). In this experiment too the asymmetric nature of the assay is utilized to demonstrate that the breast cancer cells migrate much less vigorously into the lower collagen layer which does not contain fibroblasts (Figure 6B).

## Discussion

The transition from the non-invasive to the invasive phenotype marks a fundamental change in tumor progression since tumor cell invasiveness is a pre-requisite for successful metastasis to distant organs [1]. Therefore, the understanding of the molecular mechanisms that govern the shift from the non-invasive to the invasive phenotype, and the identification of potential drugs able to inhibit tumor cell invasiveness are of great interest.

While animal experiments provide much data concerning tumor progression, they are inherently expensive and slow. In addition, in-vivo it is difficult to study molecular mechanisms due to the complexity of the environment and due to the variability of individual animals. Thus, the development of cheap quantitative in-vitro models that mimic as closely as possible the in-vivo situation yet afford precise control of experimental conditions is of prime importance for the study of the molecular mechanisms that regulate tumor progression. Like many other invasion assays, the 3D assay system we have devised relies upon the study of the behavior of tumor cells in gels containing ECM components in the presence or absence of stromal cells. The main advantage over previous assays results from the initial layout in which the tumor cells are placed as a monolayer between upper and lower gels. This confers an asymmetric nature to the assay since the composition of the upper and lower gels can be varied independently. This means that experiments designed to compare the effects of micro environmental factors can be examined in a single assay system.

An additional advantages is the existence of a sharply defined origin from which invasion begins. This means that the results of experiments are easily quantifiable. Additionally, the whole cell population is monitored which is of particular importance for the characterization of subpopulations of tumor cells and constitutes a major advantage over Boyden chamber styled assays. However, there are also disadvantages. The assay is relatively slow compared to Boyden chamber based assays which can generate results within hours, and is technically more complicated as it requires sectioning in a cryostat. This last disadvantage may be overcome in the future by the application of existing optical sectioning tools and appropriate software [21].

To demonstrate the usefulness of this assay we determined how inhibition of Loxl2 expression affects the invasiveness of cancer cells. Loxl2 is a known promoter of tumor cell invasiveness [13]. Loxl2 over-expression affects the degradation of the transcription factor snail resulting in the inhibition of the expression of E-cadherin, a known marker of epithelial to mesenchymal transition and a key regulator of tumor cells invasiveness [18,19,22]. However, this is probably not the only mechanism by which Loxl2 modulates tumor cell invasiveness and more studies are required in order to understand the mechanisms by which Loxl2 enhances tumor cells invasiveness. We have inhibited the expression of Loxl2 in several types of breast cancer cells and in cells derived from fibrosarcoma using a shRNA species specific for Loxl2 which strongly inhibits Loxl2 expression. Inhibition of Loxl2 expression strongly compromised the invasive properties of these cells suggesting that inhibitors of Loxl2 expression may possess therapeutic value. We also took advantage of the asymmetric properties of the assay by differentially adding laminin or fibronectin to the upper or lower collagen layers in order to compare directly the effects of fibronectin and laminin on the invasive ability of breast cancer cells. Interestingly, we found that the assay provided clear evidence suggesting that both fibronectin and laminin potentiate the invasion of breast cancer cells into type I collagen containing gels, and that fibronectin is a significantly more potent inducer of the invasiveness of these cells as compared to laminin. This result suggests that breast cancer cells probably do not adhere well to type I collagen and that this limits their invasive potential. This observation opens the way for more extensive comparative studies designed to determine the effects of different ECM components on tumor cell invasiveness. Our results correlate well with studies showing that decreased production of laminin and expression of fibronectin are correlated with poor prognosis in breast cancer tumors [8,23,24], although there are also studies that report different observations [25].

We exploited the asymmetric nature of our assay to determine if normal fibroblasts such as those encountered in normal tissues surrounding tumors, as opposed to tumor associated fibroblasts, can attract invading breast cancer cells and thus promote tumor cell invasiveness. Tumor associated fibroblasts have been reported to enhance tumor invasiveness by various mechanisms [26-29]. Implantation of normal foreskin derived fibroblasts on top of a monolayer of MDA-MB-231 cells attracted tumor cells that began to invade the collagen matrix towards fibroblasts located above the tumor cell monolayer but not downwards into an empty collagen layer located below the monolayer. Here again the advantage of the asymmetric nature of the assay is clearly demonstrated since the effects can be easily seen and compared with an internal control afforded by the empty lower layer of collagen.

In conclusion, we have established a novel quantitative in-vitro 3D assay designed to study modulators of tumor cell invasiveness.

## Conclusion

The *in-vitro *invasion assay we describe here enables reliable and quantitative assessment of the invasive potential of cancer cells. Contrary to the widely used Boyden chambers assay it enables assessment of the invasive potential of the whole tumor cell population. The asymmetric nature of the assay allows easy detection of attractive or repulsive effects induced by various cell types, ECM components, or purified proteins in a microenvironment that can be fashioned to closely resemble the microenvironment encountered by migrating tumor cells in-vivo. We have used it to compare the effects of laminin and fibronectin as well as fibroblasts on the invasiveness of breast cancer cells and have shown that fibronectin is a better enhancer of breast cancer cells invasion as compared to laminin, and that secreted factors derived from fibroblasts attract invading breast cancer cells.

## Abbreviations

3D: Three dimensional; ECM: Extracellular matrix; EMT: epithelial to mesenchymal transition; Loxl2: Lysyl-oxidase like protein-2.

## Competing interests

This work was funded in part by a grant from Arresto Biosciences which has an interest in loxl2. They are not covering the article charges. I do not hold stocks or shares in an organization that may in any way gain or lose financially from the publication of this manuscript. I applied for a patent (not a result of the present manuscript) which is concerned with the pro-invasive role of loxl2. I do not have any additional competing interests.

## Authors' contributions

VB carried out the experiments described in the manuscripts, developed the technique described in the manuscript, and participated in the writing of the manuscript. GN contributed to the design of the experiments and to the writing of the manuscript. Both authors have read and approved the final manuscript.

## Pre-publication history

The pre-publication history for this paper can be accessed here:

http://www.biomedcentral.com/1471-2407/9/415/prepub
